# Reaching Latinx Communities with Algorithmic Optimization for SARS-CoV-2 Testing Locations

**DOI:** 10.1007/s11121-022-01478-x

**Published:** 2023-01-09

**Authors:** Jacob A. Searcy, Camille C. Cioffi, Hannah F. Tavalire, Elizabeth L. Budd, William A. Cresko, David S. DeGarmo, Leslie D. Leve

**Affiliations:** 1 Presidential Initiative in Data Science, University of Oregon, 203 Pacific Hall, Eugene, OR 97403 USA; 2 Prevention Science Institute, University of Oregon, Eugene, OR USA; 3 Department of Counseling Psychology and Human Services, University of Oregon, Eugene, OR USA; 4 Institute of Ecology and Evolution, University of Oregon, Eugene, OR USA

**Keywords:** Facilities location problem, Latino/a/x population, COVID-19 testing, Community-informed research

## Abstract

The COVID-19 pandemic has disproportionately affected communities of color, including Latinx communities. Oregon Saludable: Juntos Podemos (OSJP) is a randomized clinical trial aimed at reducing this disparity by both increasing access to testing for SARS-CoV-2, the virus that causes COVID-19, for Oregon Latinx community members and studying the effectiveness of health and behavioral health interventions on turnout and health outcomes. OSJP established SARS-CoV-2 testing events at sites across Oregon. A critical early question was how to locate these sites to best serve Latinx community members. To propose sites in each participating county, we implemented an algorithmic approach solving a facilities location problem. This algorithm was based on minimizing driving time from Latinx population centers to SARS-CoV-2 testing locations. OSJP staff presented these proposed testing locations to community partners as a starting place for identifying final testing sites. Due to differences in geography, population distributions, and potential site accessibility, the study sites exhibited variation in how well the algorithmic optimization objectives could be satisfied. From this variation, we inferred the effects of the drive time optimization metric on the likelihood of Latinx community members utilizing SARS-CoV-2 testing services. After controlling for potential confounders, we found that minimizing the drive time optimization metric was strongly correlated with increased turnout among Latinx community members. This paper presents the algorithm and data sources used for site proposals and discusses challenges and opportunities for community-based health promotion research when translating algorithm proposals into action across a range of health outcomes.

## Introduction

A key goal of prevention science is to support public health by delivering rigorously validated evidence-based interventions. However, a key problem of practice is promoting access to these interventions (Gottfredson et al., 2015). Current approaches often fail to engage underserved communities starting at the development of a study and rarely address barriers to reaching the intended population, which can lead to project implementation failures (Spoth et al., 2013). This failure led the Society for Prevention Research to endorse a recommendation that “A system should be in place to support planning and monitoring of client recruitment. Planning should include a careful assessment of local barriers to participation and identification of strategies to overcome these barriers” (Gottfredson et al., 2015).

In the current study, we sought to identify implementation strategies to reach Latinx communities with health and health behavior interventions to overcome barriers to access. We present a new strategy to overcome this barrier by utilizing algorithmic methods from the field of operations research as a first step to inform a community-driven process. Specifically, we aim to address the problem of selecting locations for a public health service to maximize access by Hispanic/Latin/o/a/x residents of non-urban areas. The health services provided were free testing for the virus SARS-CoV-2, which causes the disease COVID-19, and a health education intervention delivered on-site. We recognize that individuals differ on self-identification, we use the term Latinx throughout for parsimony and to be more gender-inclusive of this widely diverse ethnic population (María Del Río-González, 2021). As a demonstration, we present the use of our method as implemented in the SARS-CoV-2 testing project Oregon Saludable: Juntos Podemos (OSJP).

### Oregon Saludable: Juntos Podemos

Throughout the COVID-19 pandemic, negative health burdens have fallen disproportionately on Latinx communities, which have experienced higher infection and mortality rates (Gil et al., 2020; Webb Hooper et al., 2020). Latinx communities have also seen disparities in access to SARS-CoV-2 testing resources (Grigsby-Toussaint et al., 2021), and an array of barriers such as fears regarding immigration status, lack of protected sick time from work, lack of health insurance, limited English fluency, and access to credible health information in one’s primary language, may have reduced clinical SARS-CoV-2 testing utilization (Gil et al., 2020). Disparities in access to health services for Latinx community members are exacerbated in rural communities due to social isolation, discrimination, and increasing drive time to health services because of dwindling resources as rural populations decline (Hsia & Shen, 2011; López-Cevallos & Harvey, 2016). Testing inequities have also been linked to public health outcomes with Latinx mortality increasing with testing disparities(Dalva-Baird et al., 2021).

Reducing health disparities through more equitable access to SARS-CoV-2 testing is a central goal of the National Institutes of health Rapid Acceleration of Diagnostics for Underserved Populations (RADx-UP) program (Tromberg et al., 2020). As part of this effort, we created the OSJP program to provide access to SARS-CoV-2 testing for the Latinx communities of Oregon. At the time the project started (February 2021), structural inequities were reflected in disparities in testing access and positivity rates such that Latinx community members in Oregon reflected 28.4% of cases but only 9.8% of testing encounters (COVID-19 Race, Ethnicity, Language and Disability (REALD) Report, n.d.). A key first step to start addressing these inequities with increased testing was determining where SARS-CoV-2 testing sites should be located to best support Latinx communities.

Algorithmically, selecting potential SARS-CoV-2 testing sites can be considered a facility location problem (FLP) (Weber, 1929). FLPs refer to a broad category of problems related to the selection of optimal site locations for facilities serving a population distributed across a geographic area, based on an objective and various constraints. FLPs have been studied extensively in the health care settings (Ahmadi-Javid et al., 2017), and often consider deployment of medical facilities during the aftermath of an emergency. Recently, FLP algorithms have been applied to the COVID-19 context to propose new SARS-CoV-2 testing laboratories in Nigeria (Taiwo, 2020) and to propose additional support to specific pharmacies for improved testing access (Risanger et al., 2021).

Although the use of FLP algorithms would appear to have high translational value to the field of prevention science given its focus on delivering interventions to underserved populations, we were unable to locate any publication discussing the success or failure of the FLP approach with respect to the delivery of health services and behavioral intervention for Latinx communities. A priori, it is unclear if the site locations provided by these algorithms would be actionable for SARS-CoV-2 testing because identified locations must still agree to host testing. It is also unknown if applying these algorithms would yield better utilization of SARS-CoV-2 testing by our focal population. The aim of this paper is to address this gap by presenting an FLP proposal method within a community-informed process and provide software and guidance to other groups wishing to implement similar methods. The proposed algorithm provides an opportunity for data-informed decision-making in partnership with community-based organizations. The process of sharing information with partners to make decisions about where to provide testing services reflects specific values (i.e., reaching the Latinx community) encoded by our model’s optimization goal. After presenting the FLP method, we present a post-hoc analysis demonstrating that improved site locations as estimated by the algorithm are strongly correlated with an increase in SARS-CoV-2 testing utilization by the Latinx community in Oregon.

## Methods

At the outset of OSJP, the goal of the project was to partner with community-based organizations and county public health entities to implement recurring SARS-CoV-2 testing sites across the state of Oregon. As a first step, we identified potential counties that were both feasible to reach by our testing team and had a large percentage of Latinx residents. Second, we reached out to county public health entities to determine whether they had a need for additional testing services for Latinx community members and to identify an ordering provider for the tests. County public health partners then helped our team identify community-based organizations who predominately serve local Latinx communities in each county. After establishing these partnerships, we provided each county with a report of site proposals from the FLP optimization algorithm. Final sites were selected in collaboration with our community partners. These sites hosted a series of SARS-CoV-2 testing events where the number of Latinx participants were recorded. We analyzed this observational data with a multi-level linear model to understand the effects of our optimization metric on overall participant turnout. We provide details of each step in the following sections.

### Site Location Identification Through Optimization

#### Assumptions for Objective Selection

Generically, facility location problems optimize a specific objective given several constraints. Example objectives include maximizing profit, minimizing the maximum distance to a facility, or minimizing the average distance to a facility. Example constraints include fixed maximums on the overall volume that can be served by a facility, a limit on the number of facilities, or requirements that demand is satisfied by the nearest facility. These algorithms make specific site proposals from a list of potential sites. Throughout the paper, we will refer to these as proposed sites and potential sites, with actual testing locations referred to as just sites*.*

Our goal was to best serve the Latinx community in each county with a fixed number of sites that had been pre-negotiated with county officials and community partners. In an analogous study investigating distribution of anti-viral medication, a person’s willingness to travel to obtain the medication was estimated to decline rapidly as distance increased (Singh et al., 2015). Under the assumption that most participants would be arriving by traveling in their personal vehicle, we decided the best way to serve the Latinx community was to minimize the average drive time from all Latinx population centers estimated from US Census blocks to that of their nearest OSJP testing location. The choice of this objective determines the algorithm’s balance between placing sites in primarily population dense areas to maximize attendance and providing equitable access across communities by placing sites near outlying areas. Minimizing transformed times, such as log-transformed times, alternative objectives such as minimizing the maximum distance, and other formulations are possible based on policy goals. The research team decided that by optimizing on average-drive time, a balance was achieved to provide access to testing in population dense areas, while not neglecting outlying populations. As discussed in the Results section, this decision tended to suggest more sites in rural communities than first considered given the number of available testing locations.

#### Specification of Algorithmic Function

Optimizing average estimated drive time with a specific number of sites per county is generally referred to as a *p* median FLP where *p* sites are selected to minimize a cost function. We started with *K* potential site locations for each county, *M* population centers, and *N* testing sites to place. Let ${X}_{j}$ denote if a site should be located at one of the $j=1,\dots ,K$ potential sites.

$$\begin{array}{l}{X}_{j}=\left\{\begin{array}{c}0,\qquad j \ has \ no \ site\\ \!\!\!1, \qquad j \ has \ a \ site\end{array}\right.\end{array}$$

For every $i=1,..,M$ population center with population ${P}_{i}$ we calculated an average drive time ${d}_{ij}$ to every $j$-th potential site, and we define ${Y}_{ij}$ to represent the site selection for a population center.

$$\begin{array}{l}{Y}_{ij}=\left\{\begin{array}{c}0, \qquad i \ is \ not \ served \ by \ j\\ \!\!\!\!\!\!\!\!1, \qquad i \ is \ served \ by \ j\end{array}\right.\end{array}$$

We then created a constrained objective function to minimize the average drive time of each participant.

$$L=\underset Y{min}\sum_i^M\sum_j^KP_id_{ij}Y_{ij} \qquad s.t.$$

 

$$\overset k{\underset j{\sum x_j}}=N$$

 

$$\sum_j^KY_{ij}=1\forall i=1,...,M$$

$$\sum_jY_{ij}- \ X_j \ \leq \ 0 \ \forall \ i \ =1,...,M; \ \forall \ j=1,...,K$$

The constraints enforced are that there are only $N$ testing sites, that each population site is served by only one testing site, and that population centers must be served by proposed sites only. We implemented this optimization problem using the operational research tools package OR-Tools (Perron & Furnon, 2019) in Python and utilized the COIN or Branch and Cut solver (CBC) (Forrest et al., 2020), an open-source mixed integer linear programming solver, to find near optimal solutions to the above problem. Utilizing this framework requires gathering several data elements from various sources. These elements define our population centers, potential sites, and estimated drive times, and are described in the next section.

#### Model Parameterization

Below, we provide our methods for determining parameter estimates for population centers (${{\varvec{P}}}_{{\varvec{i}}}$), potential site locations (${{\varvec{K}}}$), and estimates of the driving time between each population center and potential site (${{\varvec{d}}}_{{\varvec{i}}{\varvec{j}}}$).

##### Population Centers

Population centers were identified by utilizing GIS and demographic data provided by the US Census, and accessed with the python package *censusdata* (Leider, n.d.). The smallest geographic area publicly available is a block group where we retrieved census variable *B03002_012E*, which yielded an estimate of the total Latinx population in a given block group from the American Community Survey 5-year 2019 data (Bureau, n.d.-a). GIS data to determine the locations of each block group were retrieved directly from the Census GIS Tiger database (Bureau, n.d.-b). We utilized the geographic centroid and the population for the target demographic to define each population node.

##### Potential sites

At the time of site selection, SARS-CoV-2 community testing sites were often held as events in existing spaces that were accessible by the public and available for use by public or community entities. Potential locations were gathered from the OpenStreetMap database (OpenStreetMap contributors, 2017), which provides information on streets and locations. For potential sites, we gathered all locations within a target county tagged with any of the following attributes: parks, parking lots, schools, college, social_facilities, conference_centers, marketplaces, and places_of_worship.

##### Estimated Drive Times

Drive times were estimated using the python package OSMnx (Boeing, 2017). OSMnx utilizes the OpenStreetMap database to retrieve surface roads and build a graph with nodes at intersections connected by edges weighted by road distance divided by the associated speed limit. ${d}_{ij}$ is then calculated in parallel with each process estimating the shortest drive time on the street graph between the node geographically closest to the center of a target block group and the node geographically closest to the potential site location.

### Leveraging Algorithm-Provided Sites in the Community Partner Site Selection Process

Eight of the nine partner counties were provided a list of optimized potential sites. The number of sites used in the optimization varied by county and was determined by community needs as determined by county public health entities. One county had existing testing locations that were utilized directly. When there was uncertainty in the number of potential sites, several optimizations were performed and shared with the community partners in each county. Each list was shared with a statement of the model’s limitations:

This model focuses entirely on geographic locations, and has no knowledge of access limitations, or other factors that would impact site selection. Sites nearby each proposal should also be considered as alternatives, as they may provide practical benefits with negligible impact on the optimization objective. It is also important to note that the geographic center of census tracts may not accurately represent the population centers.

**Fig. 1:**
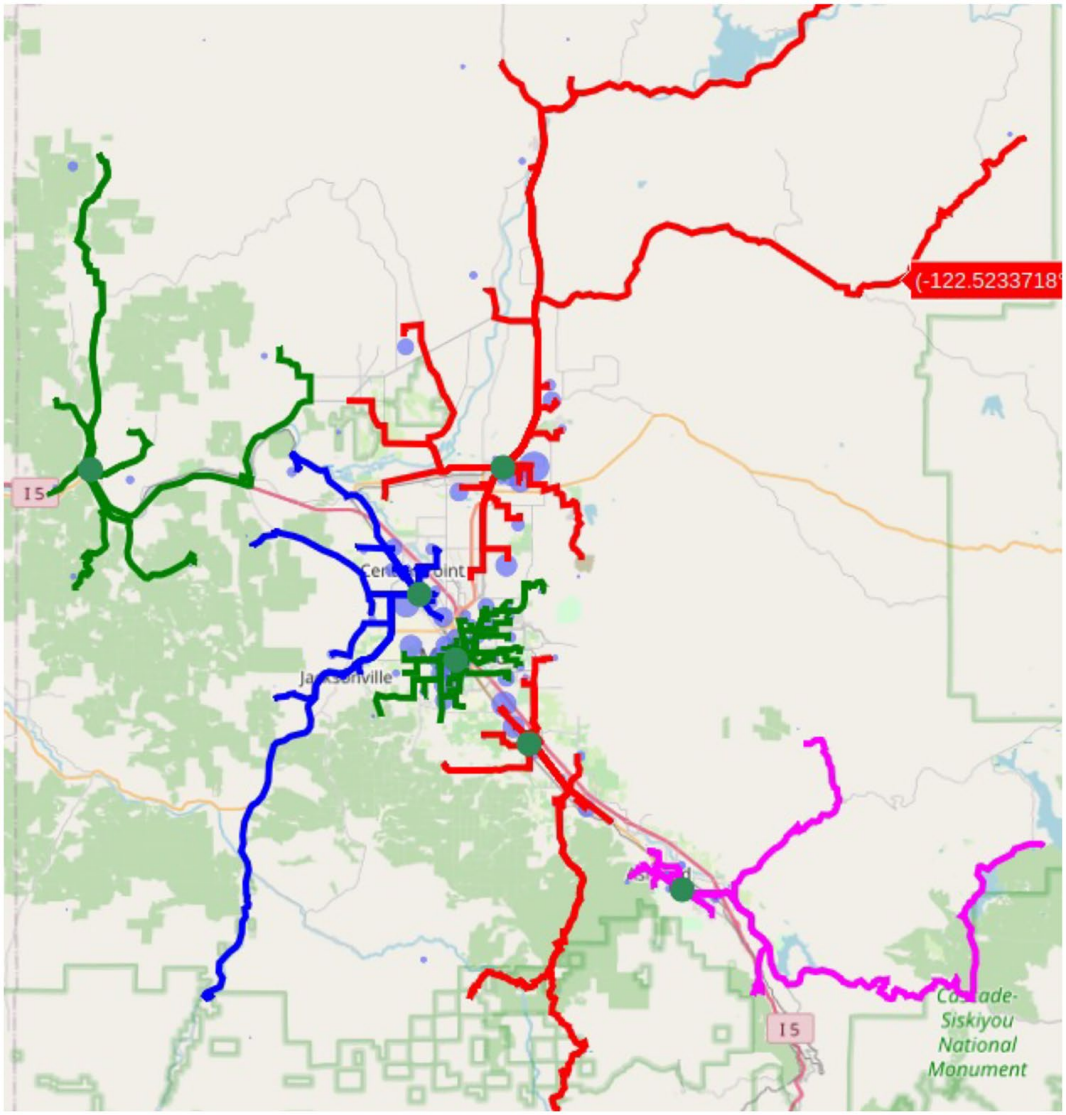
Example summary map for proposal reports

Following this warning, proposed sites and the block group centers assigned to each proposal were listed and displayed as a map such as that presented in Fig. 1. Following the generation of optimization reports, the OSJP Community Engagement Team then shared reports via email and zoom meetings generated with community collaborators to obtain feedback, and sites were either accepted, modified, or rejected. Email records and meeting minutes were analyzed to assess reasons for accepting, modifying, or rejecting algorithm-identified sites.

### SARS-CoV-2 Testing Events

Each selected site held a series of regularly occurring SARS-CoV-2 testing events. In total, there were 461 events at 56 sites in the present analyses which include data from February through September 2021. Thirty-seven sites had recurring bi-weekly events on the same day of the week and same time (except in cases of cancellations due to extreme weather, etc.). In addition to the sites with recurring events that were selected in the initial process described above, special events were held in collaboration with community partners. These were typically 1- or 2-day event that coincided with events where a large proportion of Latinx community members were expected to attend, such as Mexican Consulate Mobile events, community vaccine events marketed to Latinx community members, and other community events (e.g., concerts, markets, etc.). Participants self-identified as Latinx on the testing intake form prior to sample collection.

### Predictive Validity Evaluation

Following the implementation of testing events, we evaluated the predictive validity of the optimization metric, estimated average drive time. This quantity was calculated for all sites, not just those identified during the FLP selection process.

#### Measures

##### Number of Latinx Community Members Tested (Dependent Variable)

The criterion outcome was the number of completed SARS-CoV-2 diagnostic samples collected from Latinx individuals per testing event.

##### Average Drive Time (Independent Variable)

Average-drive time was calculated using the above data sources and methods. This was estimated from all census blocks within a 45-min drive of a testing site. This is a slightly different approach than during the optimization, where all population centers were assumed to be assigned to the nearest testing center. This conservative cutoff was required for us to utilize additional events in our analysis, particularly Mexican Consulate events which could occur in counties without other sites. Otherwise, all census blocks in the county would be assigned to one event regardless of their distance.

##### Event Type (IV)

There were several categorical classifications for distinct event types including sites participating in a randomized control trial, belonging to either a culturally tailored outreach intervention for Latinx communities called *Promotores de Salud* coded 1, or a business as usual (BAU) event coded 0 (see authors blinded for details on the randomized control trial). Vaccine sites were coded 1 if registration was offered or vaccines were administered, else the site was coded 0. Other testing events included other community partner event, coded 1 or else 0, and Mexican Consulate events coded 1 or else 0.

##### Community Characteristics by Census Tract (Covariates)

Five site-level census tract variables were matched to the testing site address with geocoded X–Y coordinates for latitude and longitude. The census tract data were matched to site addresses using FIPS geocoding. Covariates included estimated count of *Latinx populous* (number of Latinx residents/100 residents), *nativity* (number of U.S. born/100), *median age*, and *income inequality* measured with the Gini index ranging from 0 (0%) to 1 (100%), with 0 representing perfect equality and 1 representing maximal inequality.

##### Weekly SARS-CoV-2 Transmission and Cases by County (Covariates)

Two time-varying covariates potentially affecting testing rates were extracted from online databases (Centers for Disease Control and Prevention, n.d.; The New York Times, n.d.). SARS-CoV-2 transmission rate was measured by the total number of new weekly cases per county, and the total county population vaccination coverage measured as the percent of completed series of vaccinations. The weekly number of new cases was log-transformed to help meet the assumption of homogeneity of variance among predictors. Both the weekly cases and vaccine coverage were lagged by 1 week to meet time-ordered causal assumptions.

#### Analytic Strategy

A regression model was developed to test the predictive validity of the optimization metrics. A generalized linear mixed model was used to address both the repeated event data per site and the count data outcome. The resulting Poisson mixed model was fit to the log of the expected number of Latinx tests collected utilizing Eq. 1.

1 $$\begin{array}{c}Log{\lbrack E(Latinx\;test\;count)\rbrack}_{ij}=\gamma_{00}+\gamma_{01}{(average\;drive\;time)}_j+\gamma_{02}{(Latinx\;populous)}_j+\\\gamma_{03}{(nativity)}_j+\gamma_{04}{(income\;inequality)}_j+\gamma_{05}(median\;age)_j+\gamma_{06}{(vaccine\;site)}_j+\\\gamma_{07}{(Promotores)}_j+\gamma_{08}(OPE)_j+y_{09}{(Mexican\;Consulate)}_j + \gamma_{10}{(lagged\;\log\;of\;new\;cases)}_{ij}+\\\gamma_{20}{(lagged\;vaccination\;coverage)}_{ij}+u_{0j}+u_{1ij}+u_{2ij}+r\end{array}$$

where, the log test count was the expected probability of a sample test collected for the *i*th event number for the repeated testing events at the *jt*h site. To test the predictive utility of the optimization algorithm, the expected test sampling Latinx count was regressed on the level 2 predictor *γ*_01_(average drive time) _*j*_ controlling for site level census data characteristics *γ*_02*j*_ − *γ*_05*j*_ (tract nativity, income inequality, and median age), and for site classifications *γ*_06*j*_ − *γ*_09*j*_ (vaccine event, Promotores event, Oregon Health Authority event, and *Mexican Consulate event*). Two level 1 time-varying covariates were weekly cases recorded in the prior week *γ*_10*ij*_ and the county vaccine coverage in the prior week *γ*_20*ij*_, plus random residual terms for the predicted model (*u*_*0*_), level 1 covariates (*u*_1*ij −*_* u*_2*ij*_), and level 2 covariates (*r*).

## Results

### Community Partner Feedback on Algorithm Proposed Sites

Reports delivered to community collaborators contained 33 utilized site proposals. In three counties, the expected number of sites was initially uncertain, because of this we provided these counties with two optimizations, one for four sites and one for six sites. If more than four sites were selected as part of the initial selection process, then proposals from the six-site optimization were considered utilized; otherwise, the proposals from the four-site optimization were considered utilized. Of the 33 proposals, only three were able to be utilized directly; however, 24 were able to be relocated to a nearby alternative site and six were considered untenable. Of the six that were untenable, one site was already offering SARS-CoV-2 testing, one was in a wilderness area, and four were located in small remote towns where access was not possible due to travel time or lack of approval to use the proposed or alternative sites. Table 1 summarizes the proposal utilization by estimated drive time between the proposal and the replacement site. Most selected sites were placed within a 5-min drive time of the algorithm proposed locations. In addition to the algorithm proposed sites, the community partners also considered additional sites from their experiences and existing contacts. This resulted in an additional 13 sites being added with one county not utilizing site proposals. A full description of the site selection process by county is summarized in Table 2.

**Table 1:** Used algorithm proposals by distance

	**Sites**	**Count**
Proposal used as site	C2_4, C3_1, C8_2	3 (9%)
Proposals with sites located nearby with a 5-min drive	C1_2, C1_5, C2_5, C2_1, C2_2, C2_6, C3_2, C4_1, C4_2, C6_1, C6_2, C7_3, C8_1, C9_1, C9_4, C9_6	16 (48%)
Proposals with sites located nearby with a 5–10-min drive	C1_4 C2_3, C7_	3 (9%)
Proposals with sites with > 10-min drive	C1_3, C4_4, C7_2, C7_1, C7_4	5 (15%)
Rejected proposals	N/A	6 (18%)

**Table 2:** site selection notes by county

County	Reasons for moving sites
1	The CBO and CPH provided site recommendations in the communities identified by the algorithm. Reasons for not using proposals included sites being unwilling to hostesting or insufficient facilities. Two local Catholic churches who collaborated closely with OSJP’s partner CBO and the local school districts provided the final sites.
2	The CBO and CPH provided site recommendations in the communities identified by the algorithm. One proposal was retained, but was located in a community where the CBO was uncomfortable operating. Reasons for site changes included strong CBO relationships at other sites and feedback that proposals were not frequented by community members. We partnered with local schools and one Catholic church.
3	At the beginning of the project, CPH had agreed to collaborate on providing testing services, however, became unresponsive. Furthermore, the CBO communicated that they would not support events as sufficient testing existed in the county. We selected one site at a Catholic church to coincide with the end of Spanish mass.
4	We were unable to identify a CBO partner in County 4. The CPH was a willing collaborator but had a lack of knowledge about the Latinx community. Reasons for site changes included insufficient facilities and two churches that were unwilling to provide on-site testing. One clinic was adopted by the suggestion of the CPH. Other sites were selected with the local school districts and a park proximal to a proposal.
5	Members of the research study team were in County 5 and had strong working knowledge of the community and strong collaboration with the local CBO and CPH Latinx outreach team. Sites were pre-determined by these partners without algorithm use. Sites were at schools and Catholic churches with Spanish mass.
6	The CBO partner and CPH in County 6 provided site recommendations in the communities identified by the algorithm. Reasons for not using site-specific proposals included insufficient facilities and inability to determine whether an operable site existed. Final sites were a community center recommended by the local CBO and a school that was contacted after a CBO proposed church was unable to collaborate.
7	The CBO and CPH provided limited site recommendations in the communities identified by the algorithm. Our study team identified that the proposals had either insufficient facilities or were at sites that were not interested in collaborating. Final sites were comprised of schools and Catholic churches with Spanish mass.
8	The CPH provided site recommendations in the communities identified by the algorithm. One proposal at a school was retained. One proposal was rejected because of access difficulties, and was described by partners as a highly rural, White community. The remaining proposal was replaced by a proximal site.
9	The CBO and CPH provided specific site recommendations. Additionally, a study team member had strong ties to the Latinx community. Only some sites were in the communities identified by the algorithm. Reasons for site changes included insufficient facilities and inaccessibility. Final sites were comprised of one school, a community college, four places of employment, and one community events center.

*CBO* community-based organization, *CPH* county public health

During community collaborator meetings and the process of establishing site contracts, challenges emerged with some of the algorithm-identified sites. Themes included (1) locations that were not appropriate for testing or Latinx community members (2) facilities challenges, (3) site approval challenges, and (4) small population estimates of Latinx community members. Related to appropriateness, there were several sites that were invited to collaborate as testing sites who were not interested in providing testing at their location. There were also sites that were deemed by community members as being in places that Latinx community members were unlikely to frequent (e.g., churches that did not have Spanish services or did not value SARS-CoV-2 testing, libraries perceived by community partners as being under-utilized). Additionally, some early site partners who were agreeable to testing expressed aversion to having advertising materials primarily in Spanish or a focus on outreach specific to the Latinx community, so our study team identified alternate sites. In turn, we were advised by community partners to reach out to schools and churches. In our outreach to schools, the school districts typically provided guidance on which schools in their district had a greater number of Latinx students or selected school sites for our team to use based on the school district’s convenience. While our study team initially reached out to a diverse set of church denominations, anecdotally, when requesting testing sites, we found that Catholic churches were generally accepting of on-site testing and had well-attended Spanish services, demonstrating potential to reach the Latinx community.

**Fig. 2:**
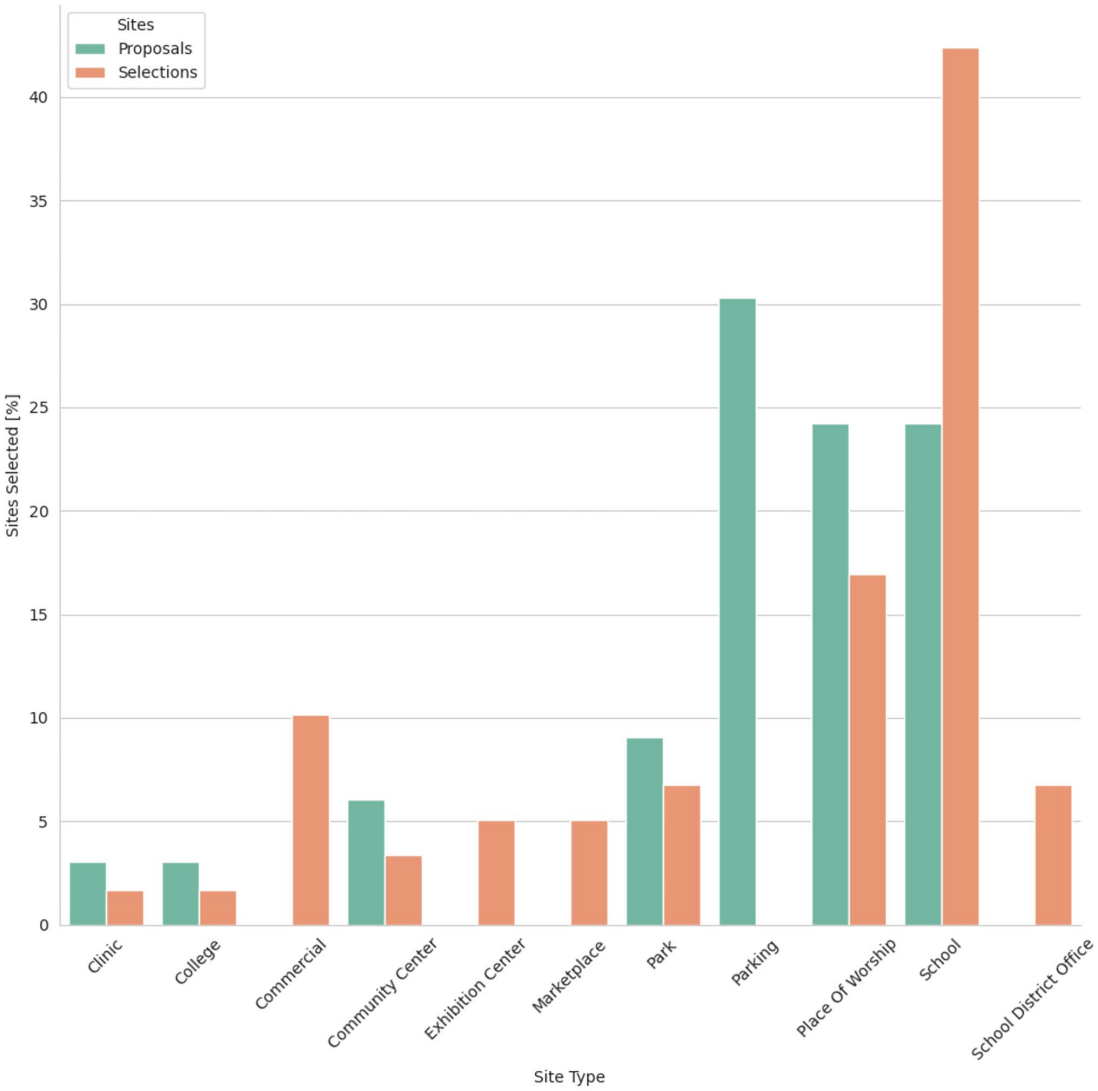
Site types for selected and proposed sites. Parking lots are common and yield good model proposals but were never selected by the community process. Schools and alternative sites not considered by the model such as commercial workplaces were preferred

A frequently raised issues with the proposed sites was a lack of facilities, such as restrooms for staff, or access to a parking lot that would be suitable for drive-up testing. While potential sites included parking lots, these were often not places where our study team would be permitted to invite additional car traffic. A breakdown of proposed and selected sites is shown in Fig. 2. For efficient use of resources, our specific objective function, minimizing drive time, often led to a few site proposals that served very rural populations. This was particularly true if the number of sites that were optimized for was large, and where small populations existed far from cities within a county. These proposals were in trailhead parking lots, or in small resort communities. One site meeting this criterion was kept but was abandoned after one event due to no turnout. The study team concluded that providing testing sites in these very sparsely populated areas was an inefficient use of resources.

### Analysis of Testing Utilization by Drive Time

Initial evaluation of the Poisson mixed model indicated predicted values were over dispersed (dispersion ratio = 2.27, *χ*^2^ = 1014.6, *p* < 0.001) and had a zero-inflation ratio of 0.60 (probable underfitting). To address this, a zero-inflated negative binomial mixed model, a special form of the Poisson (Nussbaum, 2014), was estimated in R 4.1.1 with the GLMMadaptive package (Rizopoulos, n.d.). Predictors were scaled to a variance of 1 for model convergence.

We found predictive utility of the optimization algorithm. Results for the effect of average drive time are presented in Table 3. Incident rate ratio (IRR) values > 1 indicate greater likelihood of a Latinx community member receiving a SARS-CoV-2 test, and values < 1 indicate a lower likelihood. Controlling for census characteristics and event type (which includes an outreach intervention), average drive time (scaled in the regression to 7-min units) was associated with 34% less likelihood of a Latinx individual getting tested per event. Conversely, the lower the average drive time, the greater the likelihood. Reduction in drive time represented a medium effect (*d* = 0.45). Also of note, partnering with Mexican Consulate events was highly effective for reaching the Latinx community. Several other factors such as recent spread of COVID-19 cases and outreach interventions were also found to be significant. These results are consistent with the initial findings of the OSJP clinical trial (Degarmo et al., 2022). Our analysis differs from the previous analysis, by the addition of the average distance measure and additional site types such as the Mexican Consulate events.

**Table 3:** Zero-inflated negative binomial mixed model of Latinx test samples regressed on predictors (*N* = 56 sites, 461 events)

Fixed effect	IRR	[0.78 – 1.55]
Average drive time	0.66***	[0.55 – 0.79]
Latinx populace	1.27*	[1.01 – 1.60]
Nativity	0.87	[0.72 – 1.06]
Income inequality	0.93	[0.79 – 1.10]
Median age	1.20*	[1.00 – 1.44]
Vaccine event	2.35***	[1.64 – 3.36]
Promotores event	2.14***	[1.45 – 3.16]
Other partner events	1.31	[0.76 – 2.24]
Mexican Consulate event	8.09***	[3.57 – 18.37]
Time varying covariate		
Lag county cases PC	2.00***	1.60 – 2.48
Lag vax coverage	0.87	0.74 – 1.02
Model fit		
Log likelihood	− 891.16	
AIC	1822.32	
BIC	1862.82	

*IR* incident rate ratio, *PC* per capita

^***^p < 0.001, **p < 0.05, *p < 0.05

## Discussion

The goal of this study was to assess the practical utility of an algorithm that identified the optimal location of a testing site by drive time for Latinx community members to promote SARS-CoV-2 testing. We discuss considerations for utilizing our optimization algorithm to improve services reach to Latinx community members and other underserved communities.

### Community Partner Reception of Algorithm-Generated Reports

Throughout the site selection process, the algorithm served as a springboard for discussions to help our team collaboratively identify the location of services with community partners to reach the Latinx community. The algorithm’s report anecdotally increased the likelihood that testing sites would be implemented in rural areas, where resources are often less available. In other cases, it required a re-examination of existing political realities. For example, one site was retained to serve a Latinx community where existing community partners were concerned to operate, and another site proposal was untenable due to overt hostility to the project’s aim of providing testing to the Latinx community. Having an algorithm involved in the conversation can expose blind spots in existing community networks and lead to new site considerations.

### Improving Services Utilization by Reducing Distance to Travel

Our results contribute to evidence that place-based health care is a vital tool in addressing health disparities (Dankwa-Mullan & Ṕerez-Stable, 2016), by showing that decreased average drive times, as estimated by Census and GIS data, are associated with an increase in the number Latinx participants at each testing event. This finding is intuitive but demonstrates that public data sources provide estimates that are usable for targeting locations for delivering health services and interventions.

### Limitations and Future Directions

Our study had limitations. Improvements to our site proposal algorithm could enhance usability. Several sites were considered untenable due to resource constraints (e.g., no bathroom access, not suitable for high traffic) that were not considered in the model. Our current model considered only average drive time and neglected other travel modalities such as public transit that may be particularly important in other populations particularly in urban areas (Salonen & Toivonen, 2013; Yiannakoulias et al., 2013). Our algorithm also could not account for people who may not be accurately represented in census block data, for example the unhoused (Kearns, 2012) and/or are undocumented (Capps et al., 2018; Fazel-Zarandi et al., 2018).

Our FLP algorithm also neglects the time dependence of distances which may change throughout the day as people travel to work, school, or other locations. A potential consequence was the addition of several commercial sites at place of employment by community partners as seen in Fig. 2. New data sources and algorithm development is needed to account for this dependence and consider other locations individuals may frequent. The addition of methodologies that incorporate personal sensing devices (e.g., smartphones, Fitbits) may provide a particularly novel future direction. However, by nature of our collaborations with community partners, churches, schools, and workplaces were natural choices for testing venues.

While our community partners were able to leverage the candidate sites to select proximal sites, site selection could be improved to reduce the number of site relocations required. Our model proposed sites that served very small outlier communities, and these sites were often deemed untenable because the cost and effort of running an event was considered a poor use of resources because of a low number of potential participants. This can be addressed with a common formulation of FLP algorithms that includes facility startup costs in the objective. Importantly, our model challenged status-quo assumptions during the site selection process. A key area of future research is whether algorithms should try to replicate existing decision processes or if deliberately championing different values during the site selection processes will lead to more thoughtful decisions.

We found a strong correlation between increased testing and reduced drive time. However, it is important to note that this observational analysis does not provide causal proof and does not rule out alternative explanations such as community-level characteristics. For example, individuals do not sort themselves into rural versus urban areas of residence at random, and care-seeking behavior may differ geographically based on a variety of unobserved reasons that could non-causally correlate drivetime with the likelihood of a participant seeking a test. This study did not collect individual level data on all attendees at the testing events. In addition, Latinx communities vary significantly across factors such as country of origin and primary language, which may limit generalizations drawn from this data. Demographic variation in age and gender across testing participants was also not considered. While we do not have data available on the testing team, our study was intentional about hiring individuals who were bilingual in English and Spanish and who identified as Latinx. Anecdotally, many of our testing team members were students or recently graduated from our institution, and we infer that they were mostly in their twenties.

Limitations notwithstanding, there are important contributions from our work that necessitate future research. We observed an association between reduced travel time and an increase in Latinx community members engaging in SARS-CoV-2 testing. While this provides evidence that algorithms that optimize based on drive time could be a useful tool for reaching focal populations, it is still un-proven if utilization of algorithmic proposals in collaboration with community partners resulted in significantly better optimizations compared to proposals generated by a solely community-driven process. Rigorous testing of these algorithmic proposals and their benefit is an important future direction. Doing so requires research methodologies that are designed from the outset to rigorously compare alternative optimization algorithms that are scientifically based, such as randomized comparative effectiveness or within a multiphase optimization strategy (MOST) design.

## Conclusions

We find that FLP problems are a promising algorithmic tool for supporting the delivery of health services to underserved communities, and that the information required to use them is publicly available and predictive of participant turnout. These tools, however, do not reduce the need for rigorous community engagement to work with staff at proposed sites or identify more suitable nearby alternatives if proposed sites cannot be utilized. There is significant future work to understand how these algorithms should be best formulated for use when deciding on sites with community partners. While improvements can be made to our FLP model to better reproduce the final site decisions made by the research team with our community partners, it anecdotally may be more valuable for the algorithms to champion different value systems during the consideration process. Our observational data highlight the difficulties and opportunities at providing rural services, with a strong correlation between an increase in average drive time and decreased participant turnout.
